# Participation and Yield of a Lung Cancer Screening Program in Hebei, China

**DOI:** 10.3389/fonc.2021.795528

**Published:** 2022-01-10

**Authors:** Di Liang, Jin Shi, Daojuan Li, Siqi Wu, Jing Jin, Yutong He

**Affiliations:** Cancer Institute in Hebei Province, The Fourth Hospital of Hebei Medical University, Shijiazhuang, China

**Keywords:** lung cancer, screening, Hebei province, participation rate, detection rate

## Abstract

**Objective:**

Lung cancer screening has been widely conducted in Western countries. However, population-based lung cancer screening programs in Hebei in China are sparse. Our study aimed to assess the participation rate and detection rate of positive nodules and lung cancer in Hebei province.

**Method:**

In total, 228 891 eligible participants aged 40–74 years were enrolled in the Cancer Screening Program in Hebei from 2013 to 2019. A total of 54 846 participants were evaluated as the lung cancer high-risk population by a risk score system which basically followed the Harvard Risk Index and was adjusted for the characteristics of the Chinese population. Then this high-risk population was recommended for low-dose computed tomography (LDCT) screening. And all participants attended annual passive follow-up, and the active follow-up interval was based on radiologist’s suggestion. All participants were followed-up until December 31, 2020. The overall, group-specific participation rates were calculated, and its associated factors were analyzed by a multivariable logistic regression model. Participation rates and detection of positive nodules and lung cancer were reported.

**Results:**

The overall participation rate was 52.69%, where 28 899 participants undertook LDCT screening as recommended. The multivariable logistic regression model demonstrated that a high level of education, having disease history, and occupational exposure were found to be associated with the participation in LDCT screening. The median follow-up time was 3.56 person-years. Overall, the positive identification of lung nodules and suspected lung cancer were 12.73% and 1.46% through LDCT screening. After the native and passive follow-up, 257 lung cancer cases were diagnosed by lung cancer screening, and the detection rate of lung cancer was 0.89% in the screening group. And its incidence density was 298.72 per 100,000. Positive lung nodule rate and detection rate were increased with age.

**Conclusion:**

Our study identified personal and epidemiological factors that could affect the participation rate. Our findings could provide the guideline for precise prevention and control of lung cancer in the future.

## Introduction

Lung cancer is the second most diagnosed cancer, and it is also the leading cause of cancer death in the world. According to GLOBOCAN 2020, there were approximately 2 206 771 newly diagnosed lung cancer cases and 1 796 144 cancer deaths in 2020, accounting for 11.4% and 18.0% of all new cases from cancer, respectively (1). As reported by the Chinese National Cancer Center (CNCC), with a 36.05/100,000 age-standardized incidence rate and a 28.06/100,000 age-standardized mortality rate, lung cancer was the most common cancer and the leading cause of cancer death in 2016 in China. It also showed an increasing trend in China (2). While the five-year survival rate of lung cancer was only 19.7% (3).

A series of randomized controlled trials, cohort studies, and case-control studies have demonstrated that low-dose computed tomography (LDCT) screening in a high-risk population could reduce mortality due to lung cancer (4–7). By now, lung cancer screening programs have been organized by many countries, such as the national lung cancer screening trial (NLST), National Cancer Institute Prostate, Lung, Colorectal & Ovarian Cancer Screening Trial (PLCO), and others (4, 8, 9). These trails were mainly carried out in Western countries. However, the effectiveness evaluation of lung cancer screening programs in China, in which the lifestyle is different from Western countries, is still rare.

The population-based Cancer Screening Program in Urban China (CanSPUC) was conducted in 2012. It included five type common kinds of cancer: lung cancer, female breast cancer, liver cancer, colorectal cancer, and upper digestive tract cancer (esophagus cancer and gastric cancer). Participants were invited to take a cancer risk assessment using an established clinical cancer risk score system, and those who were evaluated to be at high risk for specific types of cancer were recommended to take the appropriate screening intervention by the study design. Individuals who were found to be at high risk of lung cancer were recommended to undergo LDCT at tertiary-level hospitals.

Combined with follow-up, we aimed to assess the participation rate, screening effectiveness, and results of lung cancer screening in a high-risk population in Hebei province, China. It could provide reliable and effective data support for lung cancer prevention and control.

## Materials and Methods

### Study Population

**Figure 1:**
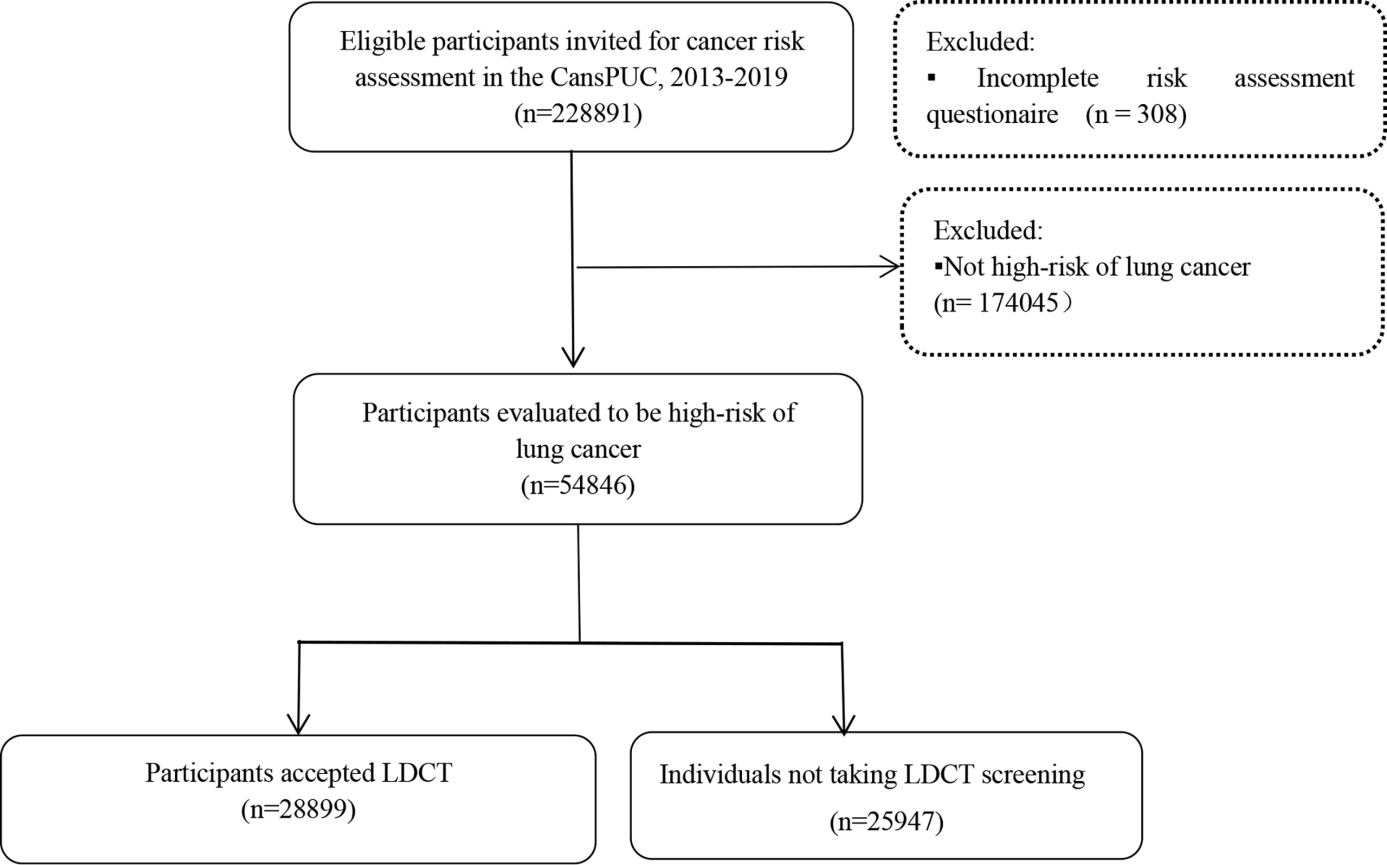
The flow chart of lung cancer screening in Hebei province, 2013-2019.

The study was conducted in Shijiazhuang and Tangshan City which are located in Hebei province (North China), and screenings took place in six tertiary-level hospitals (the Fourth Hospital of Hebei Medical University, the First Hospital of Hebei Medical University, the first Hospital of Shijiazhuang, Hebei Cheat Hospital, Tangshan People’s Hospital, and Kailuan Hospital). The participants who met the following conditions became the screening objects: (1) the residents of the program’s city; (2) residents’ age is 40-74 years old. The program used a cluster sampling method to select the screening participants. And selecting the screening participants was based on the community. The staff of the community mobilized eligible residents of the area under their jurisdiction to participate in the program. Eligible residents took part in face-to-face interviews in the selected communities. After obtaining signed informed consent, all the eligible participants were interviewed by trained staff to complete an epidemiological questionnaire and to assess their cancer risk using an established risk score system. In this study, to maximize the use of limited health resources and increase the detection rate of lung cancer, participants who were put into the high-risk groups of lung cancer were recommended free LDCT examinations in those tertiary hospitals. The present study was approved by the Ethics Board of the Fourth Hospital of Hebei Medical University. This study followed the Strengthening the Reporting of Observational Studies in Epidemiology (STROBE) reporting guideline. A flow diagram showing the recruitment of the study population is shown in **Figure 1**.

### Risk Assessment

The rationale of the cancer risk score system was based on the Harvard Risk Index (10). According to the Chinese characteristics, the risk score system included risk factors, relative risks, and exposure rates of risk factors that were adjusted. Each risk factor was allocated a score by the expert panel based on the magnitude of its association with lung cancer. The cumulative risk scores were calculated and were then divided by the average risk score in the general population to get the final individual relative risks (11). People who smoke more than one cigarette a day for more than 6 months were defined as smokers. Second-hand smoking exposure was identified in participants living with a smoker on a regular basis in the workplace or at home. The database was established by professional trained community doctors with double-entry and high-quality control to ensure consistency. The questionnaires completed every day required a random sample of 2% for re-examination, and the compliance rate of each item after the re-examination could not be less than 90%.

### LDCT Scanning

All participants undertaking the LDCT screening used the 64-section CT machine. The parameters were set as follows: (1) Scan parameters: 120 kVp and ≤30 mAs; scanning thickness: 5 mm and scanning spacing: 5 mm; the reconstructed layer thickness was 1.0-1.25 mm continuous (layer interval is 0); (2) the scanning range was from the lung tip to the costophrenic angle (including all lungs); (3) nodule measurement: Using an electronic measuring ruler to measure the maximum of the nodule length and wide diameter; (4) positive nodule: The mean diameter of solid or partly nodular nodules ≥ 5 mm, or non-solid nodules ≥ 8 mm in average diameter, or endobronchial nodules; and (5) suspicious lung cancer: A suspicious lung cancer case was identified when cases were diagnosed as suspected lung cancer or malignant lesions by senior thoracic radiologists.

### Follow-Up of Participants

All participants were followed-up by active and passive methods until December 31, 2020. An annual passive and regular active follow-up mechanism for the entire cohort population was established and carried out in our program based on the cancer registration system. Through telephone, home visits, and retrieval of medical record information from medical institutions, positive cases were actively followed-up to obtain the final diagnosis and outcome. For people with positive results, regular active follow-up was conducted by radiologist’s suggestion after the LDCT screening.

For passive follow-up, all participants who completed the questionnaire survey were matched by a personal identification number with the local cancer registration database and the all-cause mortality database in 2013-2020. The information of cancer incidence, subsite, topography, and morphology were obtained from these databases. Newly diagnosed cases of lung cancer were classified by sites according to International Statistical Classification of Diseases and Related Health Problems, Tenth Revision (codes C33 and C34).

### Statistical Analysis

The overall and group-specific participation rates by different characteristics were calculated and compared by χ^2^ test. Categorical variables were presented as numbers and percentages. The relationship of variables with participation rate of lung cancer screening were quantified by a multivariable logistics regression model with odds ratios (ORs) and their 95% confidence intervals (CIs). All statistical analyses were performed using R, version 3.4. Statistical significance was established at P ≤ 0.05 on two-sided probabilities.

## Results

### Characteristics of the Study Population

In the lung cancer screening program, 228 891 participants were recruited and had completed a risk assessment questionnaire in 2013-2019. There were 54 846 high-risk participants for lung cancer accounting for 23.96% of the total population. More women took part in the screening program, while the high-risk rate in women (43.73%) was less than that in men (56.27%). The majority of participants were between 50 and 64 years old. Most participants had junior school education level or below. In the high-risk population group, half (54.86%) had first degree relatives who had history of lung cancer, and three-quarters were smokers (**Table 1**).

**Table 1:** Characteristics of the study population and participation rates between different groups.

Variables		Numbers of questionnaire	High-risk participants (%)	LDCT screening participants (%)	Participation rates (%)	P value
**Total**		228891	54846	28899	52.69	
**Area**	Shijiazhuang	76508	14692 (26.79)	9545 (33.03)	64.97	<0.001
	Tangshan	152383	40154 (73.21)	19354 (66.97)	48.20	
**Years**	2013-2014	26171	4951 (9.03)	1899 (6.57)	38.36	<0.001
	2014-2015	33616	8317 (15.16)	2759 (9.55)	33.17	
	2015-2016	24124	5938 (10.83)	2773 (9.60)	46.70	
	2016-2017	46942	10026 (18.28)	5875 (20.33)	58.60	
	2017-2018	34942	8421 (15.35)	5204 (18.01)	61.80	
	2018-2019	39937	11788 (21.49)	7108 (24.60)	60.30	
	2019-2020	23159	5405 (9.85)	3281 (11.35)	60.70	
**Sex**	Male	109946	30863 (56.27)	13099 (45.33)	42.44	<0.001
	Female	118945	23983 (43.73)	15800 (54.67)	65.88	
**Age**	40-	30630	6142 (11.20)	3321 (11.49)	54.07	<0.001
	45-	36604	8793 (16.03)	4974 (17.21)	56.57	
	50-	40565	10737 (19.58)	5787 (20.02)	53.90	
	55-	38939	10256 (18.70)	5323 (18.42)	51.90	
	60-	42795	10901 (19.88)	5537 (19.16)	50.79	
	65-	28800	6538 (11.92)	3180 (11.00)	48.64	
	70-	10558	1479 (2.70)	777 (2.69)	52.54	
BMI*	<18.5	2090	620 (1.13)	279 (0.97)	45.00	0.002
	18.5-	91013	20662 (37.67)	10797 (37.36)	52.26	
	24-	109451	26204 (47.78)	13878 (48.02)	52.96	
	28-	26337	7360 (13.42)	3945 (13.65)	53.60	
**Educational level**	Junior school and less	140559	30411 (55.45)	13938 (48.23)	45.83	<0.001
	Senior high school	58757	15313 (27.92)	8913 (30.84)	58.21	
	College and above	29575	9122 (16.63)	6048 (20.93)	66.30	
**Job**	Technician/employee	39267	10927 (19.92)	6711 (23.22)	61.42	<0.001
	Farmer	46041	9539 (17.39)	4539 (15.71)	47.58	
	Worker	107990	27394 (49.95)	13761 (47.62)	50.23	
	Others	35593	6986 (12.74)	3888 (13.45)	55.65	
**Occupational exposure**	No	180570	27229 (49.65)	11889 (41.14)	43.66	<0.001
	Yes	48321	27617 (50.35)	17010 (58.86)	61.59	
**Fuels for heating**	clean	186540	41473 (75.62)	21258 (73.56)	51.26	<0.001
	coal	30374	11011 (20.08)	6291 (21.77)	57.13	
	Other	11977	2362 (4.31)	1350 (4.67)	57.15	
**Fuels for cooking**	Natural/liquefied gas	200943	42933 (78.28)	21436 (74.18)	49.93	<0.001
	Coal	17127	9311 (16.98)	6245 (21.61)	67.07	
	Other	10821	2602 (4.74)	1218 (4.21)	46.81	
**Smoking**	Never	168602	13262 (24.18)	8583 (29.70)	64.72	<0.001
	Smoke	52564	39853 (72.66)	19450 (67.30)	48.80	
	Ever smoke	7725	1731 (3.16)	866 (3.00)	50.03	
**Second-hand smoking exposure**	No	154473	14633 (26.68)	5457 (18.88)	37.29	<0.001
	Yes	74418	40213 (73.32)	23442 (81.12)	58.29	
**Drinking**	Never	176367	25480 (46.46)	13717 (47.47)	53.83	<0.001
	Current	46718	27227 (49.64)	14189 (49.10)	52.11	
	Former	5806	2139 (3.90)	993 (3.44)	46.42	
**History of respiratory disease**	No	186420	19796 (36.09)	6094 (21.09)	30.78	<0.001
	Yes	42471	35050 (63.91)	22805 (78.91)	65.06	
**Family history of cancer**	No	168441	18778 (34.24)	5431 (18.79)	28.92	<0.001
	Yes	60448	36067 (65.76)	23467 (81.21)	65.07	
**Family history of lung cancer**	No	190626	24760 (45.14)	8452 (29.25)	33.46	<0.001
	Yes	38265	30086 (54.86)	20447 (70.75)	67.96	

*BMI, Body mass index.

### Participation Rate for LDCT Screening

In the 54 846 participants in the lung cancer high-risk population, 28 899 undertook LDCT screening. The total participation rate was 52.69%. The screening program in Shijiazhuang (64.97%) had a higher participation rate than that in Tangshan (48.20%). Although there was a higher high-risk rate in men, the participation rate in men (42.44%) was less than that in women (65.88%). Participants aged 45-49 had the higher participation rate (56.57%), and the participation rates decreased along with the increasing age. It was found that participants with higher educational level, who worked as technical staff, had occupational exposure, never smoked, had second-hand smoke exposure, a history of lung diseases, and family history of lung cancer had relatively higher participant rates (**Table 1**).

In multivariable analysis, we found that participants who had occupational exposure had 45% higher odds of undertaking screening than other participants (OR: 1.45; 95%CI: 1.39-1.51). Smokers and former smokers were less willing to accept the screening, in which the ORs were 0.87 (95%CI: 0.81-0.92) and 0.83 (95%CI: 0.74-0.93), respectively. After adjusting for year of recruitment, study areas, married condition, Body Mass Index (BMI), drinking consumption, heating methods, and cooking fuels, we found that age, sex, educational level, occupation, occupational exposure, smoke condition, second-hand smoking exposure, history of lung diseases, and family history of lung cancer were associated with participation rate (**Table 2**).

**Table 2:** Factors associated with participation rate in lung cancer screening.

Variables		Model 1*			Model 2^#^		
		OR	95%CI	*P* value	OR	95%CI	*P* value
**Age**							
	40-	Reference			Reference		
	45-	1.24	1.16-1.33	<0.001	1.18	1.1-1.27	<0.001
	50-	1.23	1.15-1.32	<0.001	1.16	1.08-1.25	<0.001
	55-	1.31	1.22-1.41	<0.001	1.27	1.18-1.36	<0.001
	60-	1.38	1.28-1.48	<0.001	1.28	1.19-1.37	<0.001
	65-	1.34	1.24-1.45	<0.001	1.16	1.07-1.26	<0.001
	70-	1.45	1.27-1.64	<0.001	1.02	0.9-1.17	0.707
**Sex**							
	Male	Reference			Reference		
	Female	1.30	1.23-1.37	<0.001	1.40	1.33-1.48	<0.001
**Educational level**							
	Junior school and less	Reference			Reference		
	Senior high school	1.37	1.31-1.43	<0.001	1.24	1.18-1.29	<0.001
	College and above	1.67	1.57-1.78	<0.001	1.56	1.46-1.66	<0.001
**Job**							
	Technician/employee	Reference			Reference		
	Farmer	0.83	0.78-0.9	<0.001	0.70	0.65-0.76	<0.001
	Worker	0.84	0.79-0.89	<0.001	0.88	0.83-0.93	<0.001
	Others	0.95	0.88-1.01	0.119	0.88	0.82-0.94	<0.001
**Occupational exposure**							
	No	Reference			Reference		
	Yes	1.31	1.26-1.37	<0.001	1.45	1.39-1.51	<0.001
**Smoking**							
	Never	Reference			Reference		
	Smoke	0.77	0.73-0.82	<0.001	0.87	0.81-0.92	<0.001
	Ever smoke	0.77	0.68-0.86	<0.001	0.83	0.74-0.93	0.002
**Second-hand smoking exposure**							
	No	Reference			Reference		
	Yes	1.26	1.2-1.32	<0.001	1.14	1.08-1.19	<0.001
**History of respiratory disease**							
	No	Reference			Reference		
	Yes	1.81	1.72-1.89	<0.001	1.74	1.66-1.83	<0.001
**Family history of lung cancer**							
	No	Reference			Reference		
	Yes	1.50	1.41-1.60	<0.001	1.62	1.52-1.73	<0.001

*Adjusted for married condition, BMI, fuels for heating, fuels for cooking, drinking, and family history of any cancer.

^#^Adjusted for areas, year of recruitment, married condition, BMI, fuels for heating, fuels for cooking, drinking, and family history of any cancer.

### Positive Rates in Study

**Figure 2:**
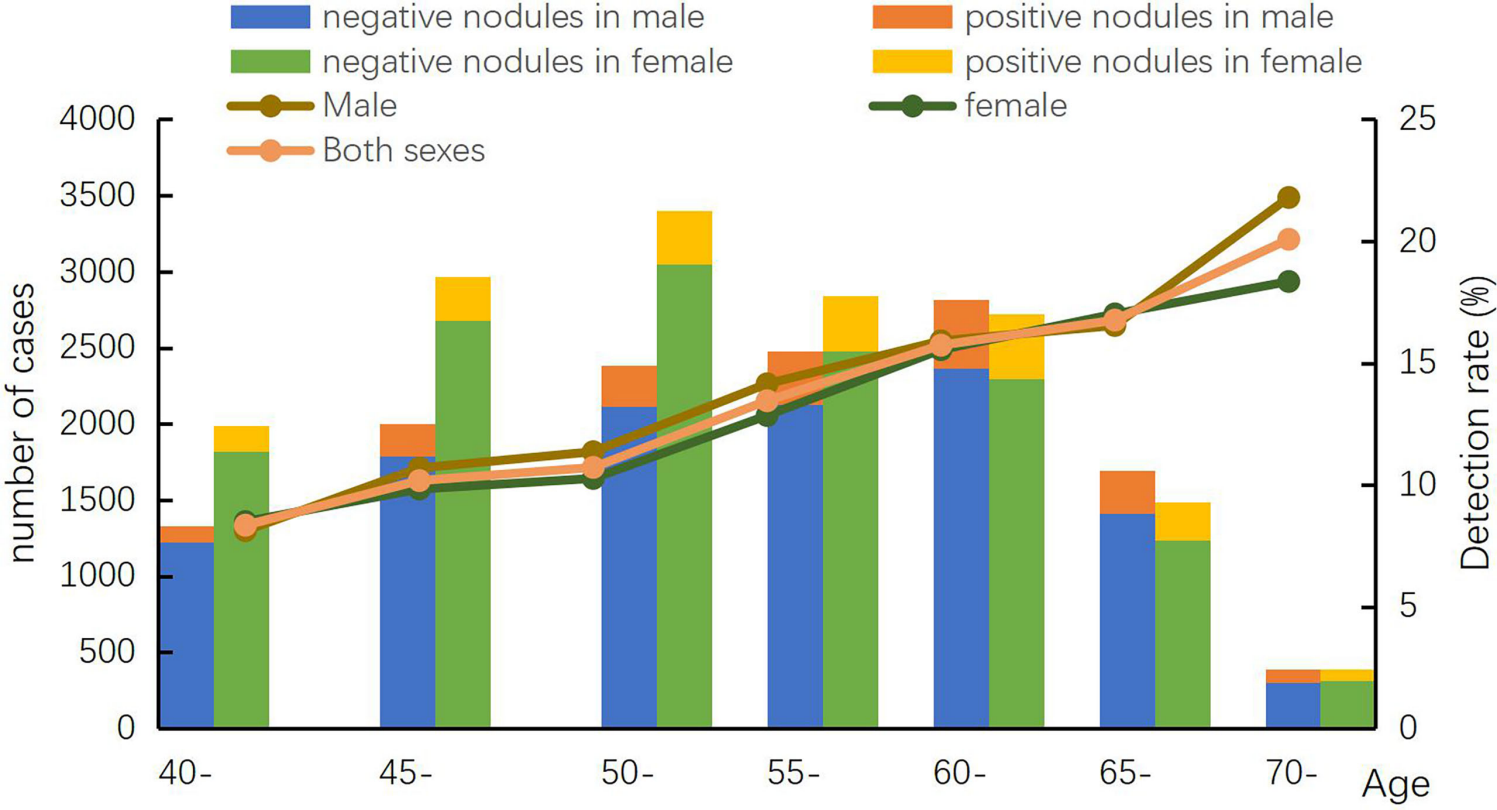
Age-specified positive nodules detection rate in Hebei province, 2013-2019.

**Figure 3:**
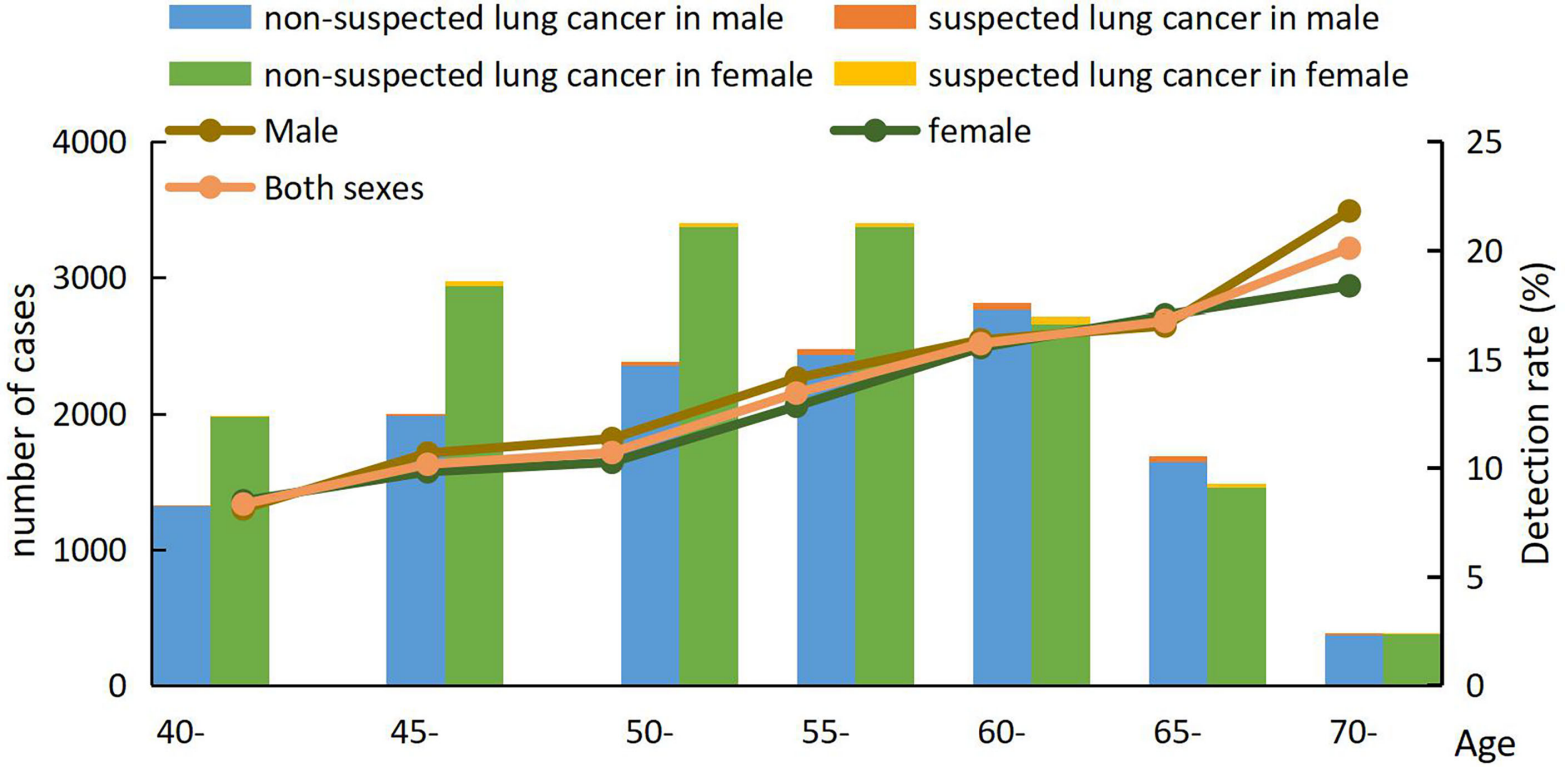
Age-specified suspected lung cancer detection rate in Hebei province, 2013-2019.

In the screening program, 3 679 positive nodules and 421 suspected lung cancer cases were detected, yielding rates of 12.73% and 1.46%, respectively. Comparing the results in different genders, the positive nodules rate in men (1757, 13.41%) was higher than that in women (1922, 12.16%). With increasing age, the positive rates gradually increased. The highest positive nodule rate was reached at 70-74 years old in both genders, which was 21.79% in men and 18.35% in women. In the positive nodules rates in ages 40-44 and 65-69, the rates in men were higher than the respective rates in women at the same age range. Along with an increasing age, the suspected lung cancer rates had an increasing trend. At 70-74 years old in both men and women, the rates reached the top which were 5.38% and 3.10%, respectively (**Figures 2** and **3**).

### Detection Rate of Positive Pulmonary Nodules

The characteristics of the nodules are shown in **Table 3**. The mean diameter of the nodule demonstrated the significant difference in benign nodule and lung cancer groups in which the median sizes were 6.00 mm and 12.25 mm, respectively. The majority of nodules were solid (83.30% in the benign nodule group and 36.63% in the lung cancer group). Non-solid and part-solid nodules accounted for 5.94% and 10.76% in the benign nodule group and 27.91% and 35.47% in the lung cancer group, respectively. A larger number of cancers were observed in the left upper (23.30%) and right upper lobes (38.07%) than in the other lobe (**Table 1**). In the lung cancer group, the proportion of nodules with stretched pleura and spiculation was higher than those in the benign nodule group.

**Table 3:** Distribution of nodule characteristics in lung cancer screening in Hebei province, 2013-2019.

	Benign nodule		Lung cancer		Total		*P* value
	N	%	N	%	N	%	
**Nodule size (mm)**							
Median	6.00		12.25		6.00		<0.001
Interquartile range	(5.00,7.50)		(8.00,17.50)		(5.00,7.50)		
**Nodule type**							
Solid	2803	83.30	63	36.63	2866	81.03	<0.001
Non-solid	200	5.94	48	27.91	248	7.01	
Part-solid	362	10.76	61	35.47	423	11.96	
Unknown	170	5.05	8	4.65	178	5.03	
**Nodule location**							
Right upper lobe	984	28.23	67	38.07	1051	28.70	<0.001
Right middle lobe	497	14.26	12	6.82	509	13.90	
Right lower lobe	816	23.41	32	18.18	848	23.16	
Left upper lobe	469	13.45	41	23.30	510	13.93	
Left lower lobe	720	20.65	24	13.64	744	20.32	
Others	20		0		20		
Unknown	119		4		123		
**Nodule’s edge**							<0.001
Spiculation	491	14.29	90	52.33	581	16.10	
Smooth	2946	85.71	82	47.67	3028	83.90	
unknown	98		8		106		
	3437		172		3609		
**Calcification**							0.008
No	3234	94.81	164	99.39	3398	95.02	
Yes	177	5.19	1	0.61	178	4.98	
Unknown	124		15		139		
**Stretched pleura**							<0.001
No	3213	94.86	125	75.30	3338	93.95	
Yes	174	5.14	41	24.70	215	6.05	
Unknown	148		14		162		

### Follow-Up Results

**Figure 4:**
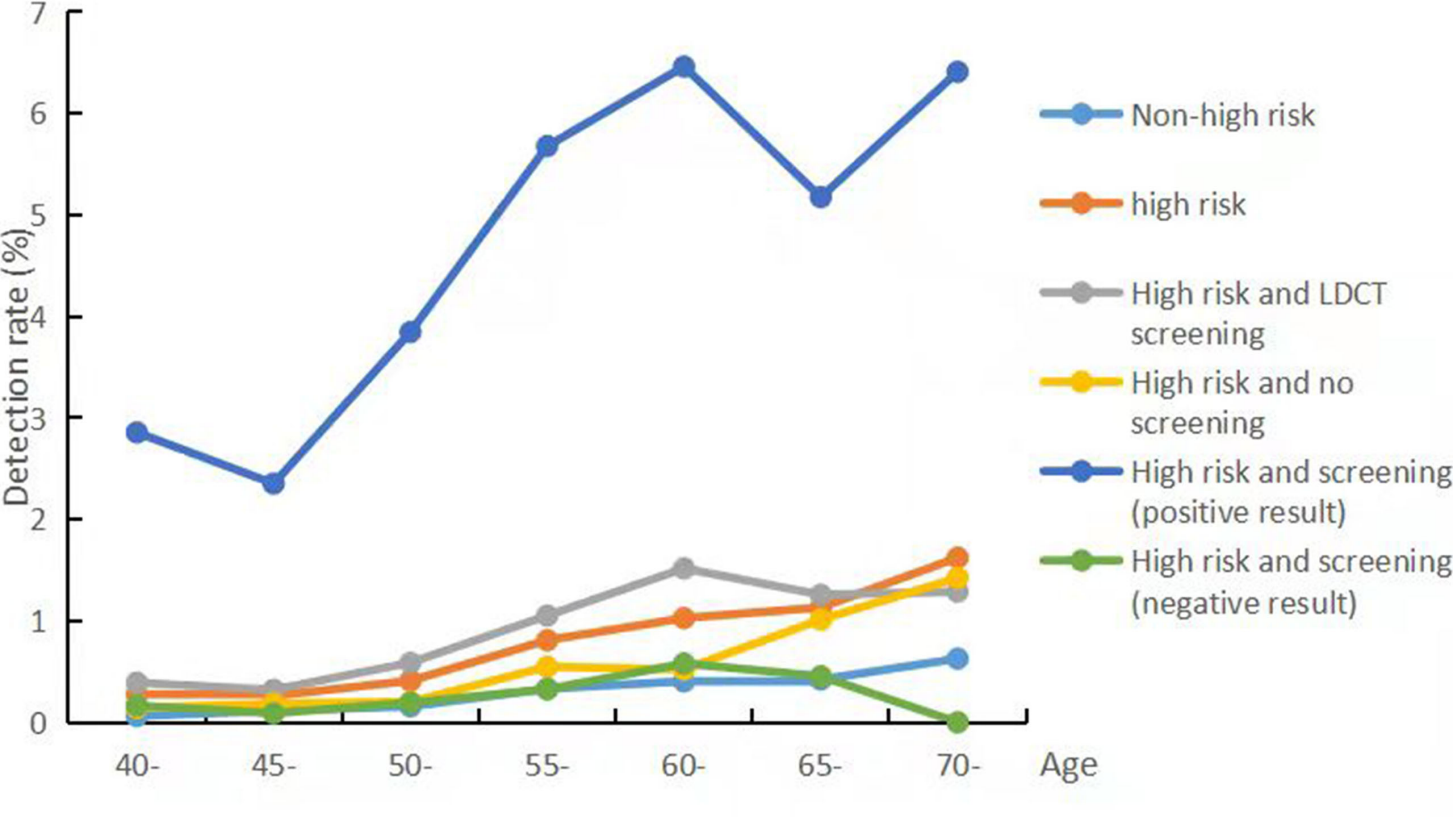
Detection rates of lung cancer in different groups, 2013-2019.

From 2013 to 2020, the median follow-up time was 3.56 years and the total follow-up time was 828 252.5 person-year. By follow-up, 257 lung cancer cases were screened in the screening group, in which the detection rate in the screening group was 0.89% and incidence density was 298.72/100,000. In the screening group, the participants with positive results (positive nodules and suspicious lung cancer) had the higher detection rate of lung cancer than participants with negative results (4.73% vs. 0.31%). In the high risk of lung cancer population, the detection rate of the screening group (0.89%) was significantly higher than those in the non-screening group (0.44%). **Figure 4** shows that the detection rates from lung cancer increased with age and those were higher in men than in women. In the screening and non-screening groups, the most common subsite of lung cancer was the upper lobe. And adenocarcinoma was the main histologic type, followed by squamous cell carcinoma and small-cell carcinoma (**Table S1**).

## Discussion

This study reported the 228 891 participants undertaking LDCT screening among a large-scale population-based screening program. This is the first study in Hebei province in China that combined epidemiological investigation, risk assessment stratification, and LDCT for participants. Although great efforts have been made by previous studies to develop effective screening, the majority of studies aimed to optimization risk scores and few were truly implemented in large-scale lung cancer screening, especially in Hebei province. The overall participation rate was 52.69% in LDCT screening among the lung cancer high-risk population. The detection rate of lung cancer in the screening group was 0.89%. And we found that the population of nodules with a relatively large mean diameter (6.00 mm vs. 12.25 mm in the benign nodule group vs. lung cancer group), non-solid, spiculation, non-calcification, and stretched pleura would more likely to develop into lung cancer. This study could provide a reliable, reasonable, and precise management strategy for lung cancer prevention and control in Hebei.

The overall participation rate was 52.69% in Hebei province. The participation rate of lung cancer screening in the high-risk population varies in different programs. It might relate to the local management and personal factors. Smoking is one of the most important factors for lung cancer and smokers were more likely to develop lung cancer (5, 12). While we found that the participation rates of lung cancer screening in smokers and former smokers were lower than that in non-smokers, in which the adjusted OR was 0.87 (95%CI: 0.81-0.92) and 0.83 (95%CI: 0.74-0.93), respectively. In smokers, the aversion to encountering adverse screening results might prevent test uptake (13–16). We also found that the population with higher educational level, who were technicians or employees, had second-hand smoking exposure, history of respiratory disease and family history of lung cancer had a higher participation rate. Previous studies demonstrate that the level of education was significantly positively correlated with the level of compliance with screening (17, 18). Our study was consistent with that of Henan province where people with undergraduate degrees or more had higher compliance (OR = 1.34, 95%CI: 1.24-1.44). It might be that the participants with a higher level of education, disease history, and occupational exposure have better understanding, self-health awareness, and pay more attention to self-care. In NLST and the European Dutch-Belgian Randomized Lung Cancer Screening Trial (NELSON), the compliance rate of screening reached 90% (4, 19). And some studies showed that the rates of participation were more than 50% (9, 20). While the overall participation rate was 34.86% in LDCT screening in three provinces (Zhejiang province, Anhui province, and Liaoning province) in China (21). And in the same program in Henan province in China, the overall participation rate was 40.16% which was lower than that in our studies. Different regional compliance was not at the same level, and other factors included the publicity and mobilization of the communities and hospitals involved in the program, the organization and mobilization process, and the health awareness of residents. Another survey conducted among family physicians in South Carolina in 2015 showed that most people had a knowledge gap and there were limited referrals of patients eligible for LDCT screening (16, 22). We conducted multiple training sessions for community physicians to educate them on the necessity and importance of lung cancer screening. As community physicians could influence screening uptake, issues related to penetration and educational outreach around LDCT screening to physicians should be examined. These studies confirmed that community physicians can help improve the compliance to a screening program, especially for people with low educational level and high age. Strengthening health education in the community system and improving the awareness rate of residents’ cancer knowledge will have a positive influence on the compliance of lung cancer screening.

In our study, the positive nodule rate was 12.73% in 2013-2019. After active and passive follow-up, the lung cancer detection rate was 0.89% in the screening group. The study of lung cancer screening in 2013-2017 in China showed that the positive rate of nodules detected by LDCT screening in high-risk groups of lung cancer was 11.36% (23). The detection rate of lung positive nodules reported in various provinces in China showed that Zhejiang province was the highest at 21.61%, followed by Beijing with 10.99%, Chongqing City, Yunnan province, Hunan province, and Henan province with 12.91%, 6.90%, 5.92%, and 5.87%, respectively (24–29). The one reason for the different levels in positive nodules rates is the different skill level of diagnosis of cancer in the early stage. During the implementation of the program, our province conducted multiple clinical diagnosis training sessions and unified the diagnostic standards to ensure the homogeneity of the data. Some of our findings with respect to the initial low-dose CT screening are not fully consistent with previous studies. The prevalence of lung cancer (0.89%) was at the middle of the reported range in some prior large studies [NLST, Early Lung Cancer Action Project (ELCAP) (30), International Early Lung Cancer Action Program (I-ELCAP) (31), NELSON (32), Rural China Screening Programme (RuraCSP) (33), Sone (34)[, which ranged from 0.4% to 2.7%. But it was close to the rate of 1.0% in the NELSON trial and 1.1% in NLST. This relatively low rate may be due to some combination of the following factors: participants in the program were healthier than the general population, and were younger in our study than in other studies. For example, our study included participants aged 40-74 and the NLST criteria included 55-74-year-old and heavy smoker participants. The other reason is that the definitions of a high-risk population were different. Following the NLST age entry criteria, the detection rate of our study in ages 55-74 was 1.28%. If the population only includes smokers, the detection rate will increase. It means that the risk assessment system of our study could concentrate on the high-risk lung cancer population and it could increase the screening effects.

Lung nodules can be effectively detected by LDCT. But discrimination between benign and malignant nodules, and which type of nodule had the greater probability of developing lung cancer are the medical concern (7). Among the positive nodules, 4.85% were malignant in our study, and this corresponded with other studies. In the Pan-Canadian Early Detection of Lung Cancer Study (PanCan) and British Columbia Cancer Agency (BCCA), the rates of cancer in nodules in the two datasets were 5.5% and 3.7% (35). We confirmed that the right upper lobes were the most common sub-site in lung cancer; they accounted for 38.07% of all diagnosed lung cancer cases. Among the screen-detected lung cancers, about three-quarters were adenocarcinomas. And the screening methods for small cell lung cancer and squamous cell carcinoma need to be improved. Lung adenocarcinomas are more likely to be located at the periphery of the lung. And the cancer in the lung periphery had a greater probability of being measured than central lung cancer (36). Lung cancer is most likely to occur in the upper lobe. It is a known phenomenon in non–small cell lung cancer cases and can be explained as the maximum airflow when breathing begins, mainly towards the upper right lobe bronchus. So, tobacco smoke and its carcinogenic toxins accumulates the most in the right upper lobe (37–39). Through our study, we confirmed that nodules with the following characteristics should be paid more attention to in future clinical treatment and diagnosis: larger nodule size, location of the nodule in the upper lobe, non-solid and part-solid nodule type, spiculation, non-calcification, and stretched pleura nodules (35). These nodules were more likely to develop into lung cancer.

This study has strength and limitations. The strengths were as follows: this study was population-based, and it involved a large-scale sample size. Detailed epidemiological questionnaire information was collected in a standardized manner by trained study staff to ensure the quality of the data. A sound annual passive and active follow-up mechanism for the entire cohort population was established and carried out in our program based on the cancer registration system. We obtained information regarding each participant’s cancer incidence in the study. This study has the limitation that some variables, such as smoking status and other variables, were self-reported and it might lead to misclassification. Another limitation is that follow-up work for patients diagnosed with lung cancer is still under way, therefore clinical disease information was not fully obtained. And the study population was a pre-selected high-risk population ascertained by the risk assessment system which might not represent the general population of Hebei province, and selection bias cannot be ruled out.

In summary, in this large-scale lung cancer screening in Hebei, we found that some variables, which were age, sex, educational level, job, smoker, secondhand smoking exposure, history of respiratory, and family history of lung cancer contributed to the participation rate. And the detection rate in the screening group was higher than that in other groups. Our finding may provide data support for lung cancer prevention and it is useful for optimizing screening strategies.

## Data Availability Statement

The raw data supporting the conclusions of this article will be made available by the authors, without undue reservation.

## Ethics Statement

The studies involving human participants were reviewed and approved by the Ethics Board of the Fourth Hospital of Hebei Medical University (No. 2012KY102). The patients/participants provided their written informed consent to participate in this study.

## Author Contributions

DiL and YH wrote the main text and conducted data analysis. YH designed the study. JS, DaL, SW, and JJ collected the data. All authors contributed to the article and approved the submitted version.

## Conflict of Interest

The authors declare that the research was conducted in the absence of any commercial or financial relationships that could be construed as potential conflicts of interest.

## Publisher’s Note

All claims expressed in this article are solely those of the authors and do not necessarily represent those of their affiliated organizations, or those of the publisher, the editors and the reviewers. Any product that may be evaluated in this article, or claim that may be made by its manufacturer, is not guaranteed or endorsed by the publisher.
