# Supplementary material for: Participation and Yield of a Lung Cancer Screening Program in Hebei, China
Source: Front Oncol. 2022 Jan 10;11:795528. doi: 10.3389/fonc.2021.795528 (PMC8784378; doi:10.3389/fonc.2021.795528)
Supplement: Supplementary file 1 [file Table_1.docx]

Table S1 Characteristics of lung cancer in screening and non-screening group

| **Variables** |  | **Screening group** | |  | **Non-screening group** | |
| --- | --- | --- | --- | --- | --- | --- |
|  |  | **N** | **%** |  | **N** | **%** |
| **Subsite** | Trachea | 0 | 0.00 |  | 1 | 0.34 |
|  | Main bronchus | 0 | 0.00 |  | 5 | 1.68 |
|  | Upper lobe | 79 | 63.71 |  | 154 | 51.85 |
|  | Middle lobe | 9 | 7.26 |  | 42 | 14.14 |
|  | Lower lobe | 36 | 29.03 |  | 95 | 31.99 |
|  |  |  |  |  |  |  |
| **Morphology** | Squamous cell carcinoma | 16 | 8.56 |  | 65 | 17.02 |
|  | Adenocarcinoma | 138 | 73.80 |  | 250 | 65.45 |
|  | Small cell carcinoma | 16 | 8.56 |  | 49 | 12.83 |
|  | Others | 17 | 9.09 |  | 18 | 4.71 |
